# Altered neural oscillation patterns and functional connectivity in postherpetic neuralgia: evidence from resting-state EEG

**DOI:** 10.3389/fpain.2025.1603345

**Published:** 2025-07-24

**Authors:** Fei Gao, Mengru Wang, Huibin Jia, Georgi V. Georgiev, Yi Feng, Wenxia Li

**Affiliations:** ^1^ Department of Pain Medicine, Peking University People’s Hospital, Beijing, China; ^2^ Center for Ubiquitous Computing, University of Oulu, Oulu, Finland; ^3^ School of Psychology, Henan University, Kaifeng, Henan, China; ^4^ Department of Anesthesiology, Xiyuan Hospital, China Academy of Chinese Medical Sciences, Beijing, China

**Keywords:** postherpetic neuralgia, electroencephalography, power spectrum density, functional connectivity, neural oscillations

## Abstract

Postherpetic neuralgia (PHN) is a debilitating chronic pain condition that significantly affects the quality of life, often leading to physical discomfort, emotional distress, and psychological comorbidities, such as depression and anxiety. This study aimed to systematically assess the neural oscillatory activity and functional connectivity patterns in patients with PHN using resting-state electroencephalography (EEG). Overall, 21 patients with PHN and 17 healthy controls participated in this study. Resting-state EEG data were collected and analyzed. Power spectrum density analysis was conducted to compare frequency band activity, and correlation analyses were used to examine associations between EEG power and psychological symptoms. Functional connectivity was assessed using the weighted phase lag index. Power spectrum density analysis revealed significantly higher EEG power across the delta, theta, alpha, beta, and gamma frequency bands in patients with PHN compared with controls. Correlation analyses indicated that delta and theta frequency band power were positively associated with the Beck Depression Inventory scores, whereas delta frequency band power was negatively correlated with the State Anxiety Inventory scores. Additionally, functional connectivity analysis demonstrated reduced gamma-band connectivity in patients with PHN, particularly in regions of the sensorimotor and pain modulation networks. These findings suggest that PHN is characterized by widespread hyperactivity in neural circuits, accompanied by disrupted interregional communication. These results provide valuable insights into the neurophysiological mechanisms underlying PHN and highlight potential biomarkers for developing targeted neuromodulatory treatments to alleviate pain and improve the psychological well-being of affected individuals.

## 1 Introduction

Postherpetic neuralgia (PHN) is a debilitating chronic pain condition that arises as a complication of herpes zoster (shingles) (1). Characterized by neuropathic pain that persists long after the herpes zoster rash has healed, PHN significantly reduces the quality of life of patients, contributing to physical discomfort, emotional distress, and psychological comorbidities, such as depression and anxiety (2). This condition predominantly affects older adults, with the prevalence rates increasing with age (3). Despite the availability of antiviral treatments and analgesics, PHN remains challenging to manage and its pathophysiological mechanisms are not fully understood. This knowledge gap underscores the need for more comprehensive investigations into the neural underpinnings of PHN.

Pain perception and chronic pain disorders are intricately linked to brain function and connectivity (4). The brain processes pain through complex neural networks, involving various oscillatory activities across multiple frequency bands ranging from slow delta (1–4 Hz) to fast gamma (30–70 Hz) rhythms (5). These oscillatory patterns play a critical role in sensory, cognitive, and emotional processing. Alterations in neural oscillations and functional connectivity are increasingly recognized as hallmarks of chronic pain conditions, reflecting disruptions in the balance between excitatory and inhibitory brain activity (6). Investigation of these oscillatory patterns offers valuable insights into the neural dynamics underlying PHN and potentially informs the development of novel therapeutic interventions.

Electroencephalography (EEG) is a noninvasive, high-temporal-resolution method for assessing brain activity and connectivity, making it an ideal tool for studying the neural correlates of chronic pain (7, 8). The interplay between the alpha and gamma oscillations plays a significant role in predicting the perception of nociceptive stimuli. Peristimulus fluctuations in both bands modulate sensory perception, with alpha oscillations reflecting inhibitory control and gamma oscillations indicating sensory integration (9). Previous studies have demonstrated that patients with chronic pain conditions exhibit altered power spectra and functional connectivity across various EEG frequency bands. Increased gamma power has been observed in patients with PHN, suggesting heightened cortical excitability and enhanced sensory processing (10). Gamma oscillations in the primary somatosensory cortex are directly correlated with subjective pain intensity, reflecting the cortical activity linked to pain perception rather than simply reflecting attention or arousal (11). However, research focusing on the broader spectrum of oscillatory changes in patients with PHN, particularly in the delta, theta, alpha, and beta bands, is limited. Furthermore, few studies have investigated the relationship between EEG findings and psychological disorders, such as anxiety and depression, which are commonly comorbid with chronic pain conditions (12).

Functional connectivity, which reflects the degree of synchronization between various brain regions, is another crucial factor that impacts pain processing (13). Disrupted brain connectivity patterns have been reported in various chronic pain conditions, indicating impaired communication within pain-related neural networks (14). Specifically, a decrease in connectivity within the descending pain modulatory pathways and an increase in connectivity within the ascending pain pathways contribute to persistent pain in patients with PHN (15). Understanding the differences in functional connectivity between patients with PHN and healthy individuals could provide critical insights into the neural mechanisms driving chronic pain and guide the development of targeted neuromodulator therapies.

To comprehensively assess the impact of PHN on physical and emotional health, this study utilized several standardized psychometric tools: the Visual Analog Scale (VAS), Beck Depression Inventory (BDI), State Anxiety Inventory (SAI), and Trail Anxiety Inventory (TAI). The VAS is a simple yet effective measure of pain intensity and is frequently used in clinical and research settings. During assessment with VAS, patients mark their pain level on a 10-cm line, with its endpoints representing “*no pain*” and “*worst pain imaginable*.” This tool offers a quantitative measure of subjective pain experience, which is crucial for tracking the severity of PHN over time (16). BDI is a widely used self-report questionnaire designed to evaluate the severity of depressive symptoms. It includes 21 items and covers a range of emotional, cognitive, and physical symptoms associated with depression (17). The State-Trait Anxiety Inventory (STAI) assesses anxiety using two complementary subscales: the State Anxiety Inventory (SAI), which measures situational anxiety (18), and the Trait Anxiety Inventory (TAI), which evaluates general anxiety tendencies (19).

This study aimed to systematically investigate the resting-state EEG oscillations and functional connectivity in patients with PHN. By comparing the EEG power spectral density (PSD) and connectivity patterns between patients with PHN and healthy controls (HCs), this study sought to identify the neural signatures associated with chronic pain. Additionally, the study explored the correlations between the EEG oscillations and scores from psychological assessments, including the VAS, BDI, SAI, and TAI, to elucidate the interplay between neural activity and emotional distress in patients with PHN.

We hypothesized that patients with PHN will exhibit increased power across multiple EEG frequency bands, reflecting hyperactivity in pain-related neural circuits. Furthermore, functional connectivity, particularly in the gamma band, is anticipated to be reduced in patients with PHN, indicating disrupted communication between the brain regions involved in pain processing. By addressing these hypotheses, this study aimed to contribute to a more comprehensive understanding of the neural basis of PHN and provide potential biomarkers for guiding future interventions.

## 2 Methods

### 2.1 Participants

#### 2.1.1 Patient group

Twenty-one right-handed patients [age range: 43–95 years; mean ± standard deviation (SD): 69.10 ± 11.19 years; 11 female] diagnosed with PHN were recruited from the outpatient clinic at XX. Experienced clinicians diagnosed PHN based on clinical symptoms, including medical history, characteristic scarring, and pain severity.

The inclusion criteria were age >18 years and presence of persistent pain for a minimum duration of 1 month. Patients with a history of migraine, tension headache, peripheral neuropathy, osteoarthritis, or any other conditions associated with acute or chronic pain, chronic illnesses, or neurological disorders that could potentially influence the EEG results were excluded.

Patients with PHN were not medication-free at the time of the study and were prescribed gabapentin, pregabalin, or nonsteroidal anti-inflammatory drugs. Patients who received opioid analgesics were excluded. Table 1 summarizes the demographic information, drug history, and clinical characteristics of the patient cohort.

**Table 1:** Clinical descriptions of patients with PHN.

No.	Anatomical site of pain	Pain laterality	Drug history	Pain duration (months)	VAS score	SAI score	TAI score	BDI score
1	Arm	Left	NSAID	1	5	41	36	19
2	Chest and back	Left	Gabapentin	10	7	48	55	13
3	Arm	Right	Gabapentin	1	9	53	46	4
4	Chest and back	Left	Gabapentin	15	9	44	50	39
5	Chest and back	Right	Gabapentin	2	9	44	59	24
6	Chest and back	Left	Gabapentin	29	6	50	48	8
7	Chest and back	Right	Gabapentin	2	9	47	52	20
8	Chest and back	Right	Gabapentin	2	6	49	48	2
9	Arm	Right	Gabapentin	5	8	49	46	4
10	Face	Right	Gabapentin	31	7	55	49	9
11	Chest and back	Right	Gabapentin	3	7	47	47	1
12	Arm	Left	Gabapentin	4	6	39	34	8
13	Chest and back	Left	Gabapentin	1	4	33	30	2
14	Chest and back	Left	None	8	4	48	44	5
15	Chest and back	Right	None	36	4	50	47	7
16	Chest and back	Right	Gabapentin	1	3	50	46	3
17	Chest and back	Left	Gabapentin	1	4	39	41	9
18	Chest and back	Right	Gabapentin	1	4	40	41	2
19	Chest and back	Right	Gabapentin	7	5	42	46	7
20	Arm	Right	Gabapentin	1	8	47	44	8
21	Chest and back	Right	None	7	5	45	39	15

VAS, visual analog scale; SAI, state anxiety inventory; TAI, trait anxiety inventory; BDI, beck depression inventory; NSAID, nonsteroidal anti-inflammatory drug.

#### 2.1.2 Hc group

The HC group consisted of 17 right-handed individuals (age range: 44–79 years; mean ± SD: 64.47 ± 10.04 years; 6 female).

All participants provided written informed consent before participation. This study was reviewed and approved by the Ethics Committee of XX.

### 2.2 Clinical evaluation

All participants completed the Short-Form McGill Pain Questionnaire (SF-MPQ) (20). It includes a verbal component and a VAS ranging from 0 (indicating no pain) to 10 (representing the maximum imaginable pain). Anxiety and depression levels of the participants were assessed using the STAI (19) and BDI (21), respectively.

The STAI comprises the State Anxiety Inventory (SAI) and Trait Anxiety Inventory (TAI) subscales. The SAI, consisting of 20 items, measures state anxiety in response to situational stress, whereas the TAI, which also contains 20 items, evaluates the frequency of general emotional experiences. The BDI, designed to assess depressive symptoms, includes 13 items that assess how participants felt during the past week. The responses are scored on a scale from 0 (“*not at all*”) to 3 (“*severely*”), with higher cumulative scores indicating more severe depressive symptoms. All questionnaires were administered 1 h prior to the EEG recording session.

### 2.3 EEG recording

The participants were seated in comfortable chairs in a silent, temperature-controlled room and instructed to relax, with their eyes closed. Resting-state EEG data were recorded for 10 min using NuAmps device (NeuroScan Labs, Australia). Thiry-two Ag/AgCl scalp electrodes were positioned in accordance with the international 10–20 system at Fp1, Fp2, F7, F3, Fz, F4, F8, FT7, FC3, FCz, FC4, FT8, T3, C3, Cz, C4, T4, TP7, CP3, CPz, CP4, TP8, T5, P3, Pz, P4, T6, O1, Oz, O2, A1, and A2. The EEG signals were referenced to the average of the mastoids, and the electrode impedance was maintained below 10 kΩ during the recording process.

EEG data were amplified, digitized at 1 kHz sampling rate, and band-pass filtered (0.1–100 Hz). To ensure that the participants remained awake during data collection, they were prompted to report their conscious state at 1 min intervals.

### 2.4 EEG data preprocessing

EEG data were preprocessed using the EEGLAB toolbox (v.13.0.0b) in conjunction with customized scripts in MATLAB 2013b (22). Continuous EEG signals were band-pass in the range of 1 to 70 Hz and a 50 Hz notch filter was applied to eliminate line noise. Epochs were segmented into nonoverlapping 2 s intervals. Electrooculogram artifacts were corrected using independent component analysis decomposition. Subsequently, the data were re-referenced to the average of all the available channels.

### 2.5 PSD analysis

The PSD values were computed for each channel and epoch using the MATLAB function “*periodogram*” and customized scripts, with a nonoverlapping fast Fourier transform length of 2,048. The PSD values were averaged across epochs for each participant. Subsequently, the PSD values were extracted and averaged within predefined frequency bands. These bands were classified as delta (1–4 Hz), theta (4–7 Hz), alpha (7–13 Hz), beta (13–30 Hz), and gamma (30–70 Hz).

A two-sample t-test was conducted to compare patients with PHN and HCs across all frequency bands and channels. This analysis was performed using the MATLAB function “*ttest2*.” To account for multiple comparisons, false discovery rate correction was applied at a significance threshold of corrected *p* < 0.05, utilizing the MATLAB function “*mafdr*.”

Partial correlation analyses were performed to investigate the relationship between the mean PSD value across all channels in each frequency band and the BDI, SAI, and TAI scores. Sex and age were included as covariates to control for potential confounding effects. Partial correlations were calculated using the MATLAB function “*partialcorr*.”

### 2.6 Functional connectivity analysis

Functional connectivity strength was assessed using the weighted phase lag index (wPLI), a measure designed to mitigate the effects of volume conduction (23). The wPLI was computed for each pair of channels that exhibited significant differences in the PSD values between the HCs and patients with PHN. This selection criterion was used to narrow the connectivity analysis to the brain regions that showed significant spectral changes, assuming that these areas were more likely to reflect the pathophysiological changes associated with PHN. Although this approach improves sensitivity to PHN-related effects, it may limit the generalizability of connectivity findings to the entire brain network. A customized MATLAB script was employed to calculate the wPLI using the following equation:

$$\Phi \equiv \frac{\left|E\{ℑ\{X\}\}\right|}{E\{\left|ℑ\{X\}\right|\}}$$

To investigate whether the wPLI values differed significantly between HCs and patients with PHN across frequency bands, the network-based statistic (NBS) method was applied. NBS is a nonparametric approach designed to address the multiple comparison problem arising from mass univariate statistical tests in functional networks (24). The analysis performed is described below.

First, the *T*-test was performed to compare the wPLI values between the two groups for all 325 possible channel pairs (C_26_^₂^). In the NBS framework, a general linear model was implemented (25). Second, an uncorrected threshold of *p* < 0.05 was applied to identify channel pairs with potential differences in the wPLI values, forming connected graph components. Third, the size of each connected graph component was defined based on the total number of significant channel pairs. A permutation test (*n* = 5,000) was then conducted, during which participants were randomly reassigned to one of the two groups for each permutation. The maximum cluster size from each permutation was used to construct an empirical null distribution for the largest component size. Finally, to control for multiple comparisons, a one-sided family-wise error corrected *p* value (*p* < 0.05) was applied at the cluster level. All statistical tests were performed using the MATLAB toolbox NBS v1.2 (https://www.nitrc.org/projects/nbs/).

## 3 Results

### 3.1 PSD

**Figure 1:**
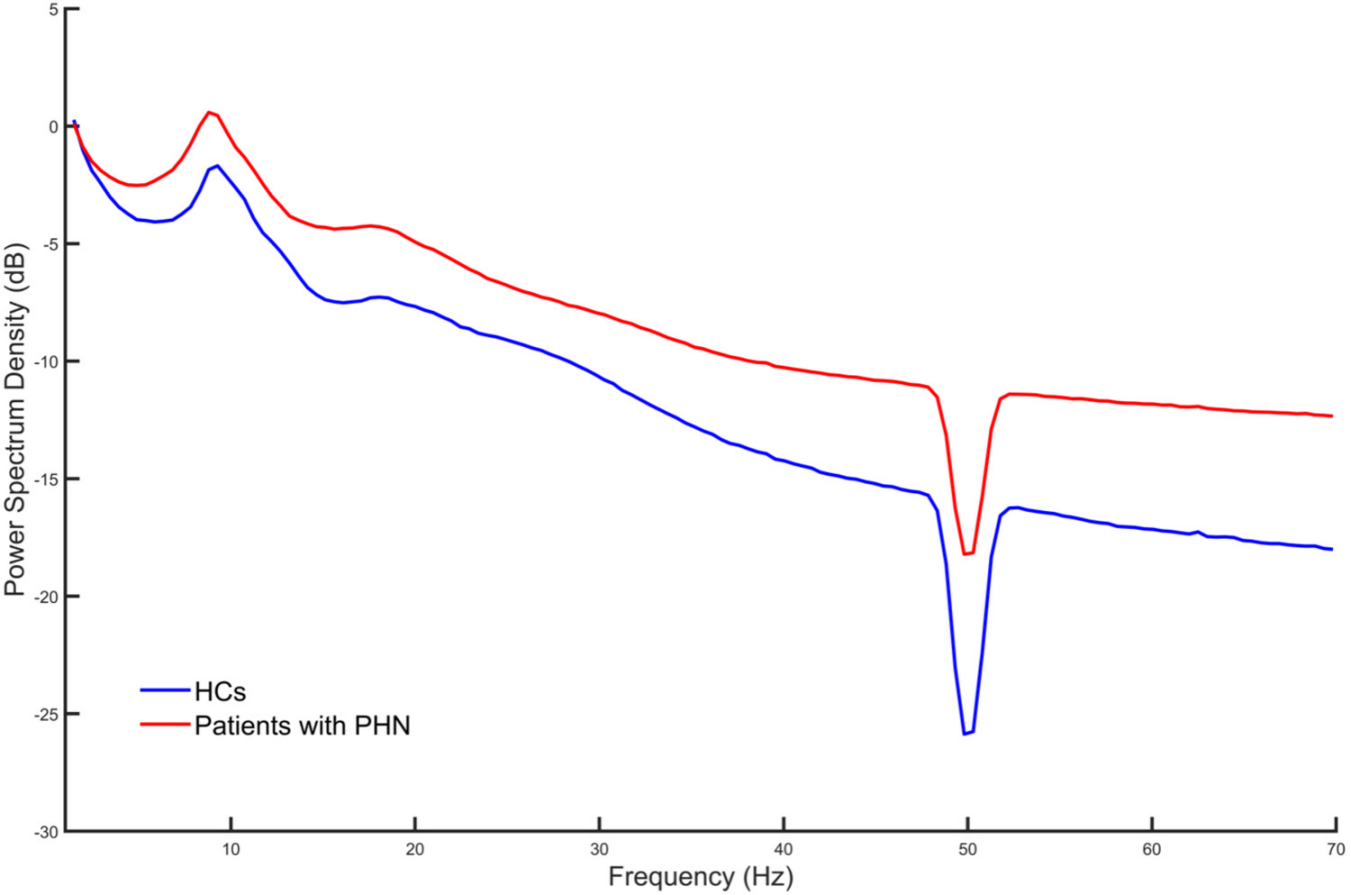
Average power spectrum density across all channels and participants in each group. HC, healthy control; PHN, postherpetic neuralgia.

Spontaneous EEG PSD within the frequency range of 1–70 Hz was computed for patients with PHN and HCs. Figure 1 indicates the averaged PSD value across all channels and participants for each group. As shown in Figure 1, the PSD values were consistently elevated across all frequency bands in patients with PHN compared with HCs.

**Figure 2:**
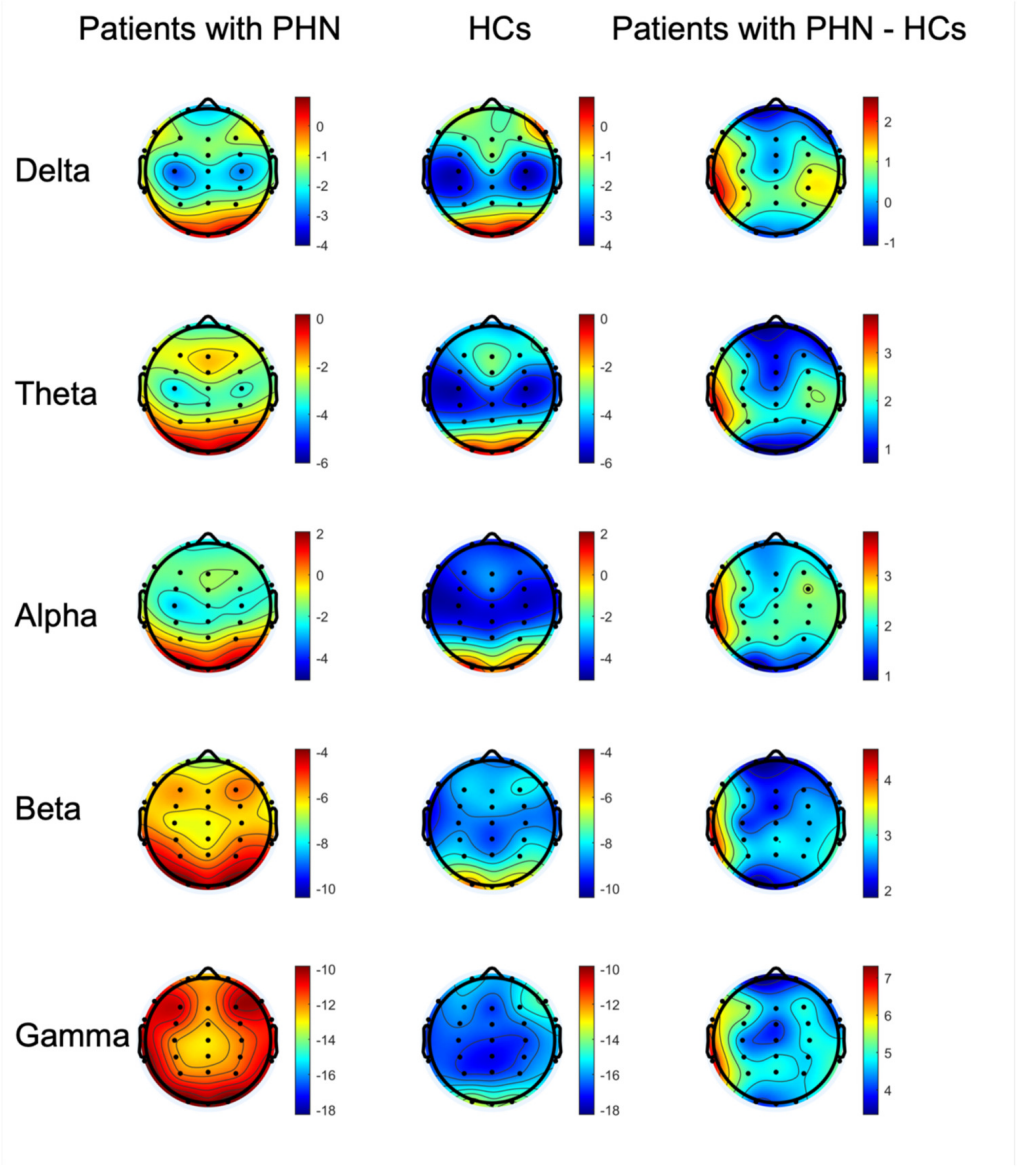
Topography of the power for the delta, theta, alpha, beta, and gamma frequency bands in the patients with PHN, HCs, and the difference in power between the two groups (patients with PHN minus HCs) (unit: dB). HC, healthy control; PHN, postherpetic neuralgia.

Figure 2 illustrates the PSD distribution on maps across each frequency band for both groups (the left and middle columns), highlighting their differences (the right column). Specifically, in the delta and gamma frequency bands, the PSD values across all channels were significantly higher in patients with PHN than in HCs (corrected *p* < 0.05). For the theta frequency band, the PSD values of 23 channels (Fp1, Fp2, F3, Fz, F4, F8, FC3, FCz, FC4, FT8, C3, Cz, C4, CP3, CPz, CP4, TP8, P3, Pz, P4, O1, Oz, and O2) were significantly higher in patients with PHN compared with HCs (corrected *p* < 0.05). Similarly, in the alpha frequency band, 23 channels (Fp1, Fp2, F3, Fz, F4, FC3, FCz, FC4, FT8, C3, Cz, C4, TP7, CP3, CPz, CP4, TP8, P3, Pz, P4, O1, Oz, and O2) exhibited significantly higher power in patients with PHN than in HCs (corrected *p* < 0.05). In contrast, for the beta frequency band, only two channels (F7 and FT7) showed significantly higher power in patients with PHN than in HCs (corrected *p* < 0.05).

The pain ratings and psychological assessment scores are presented in Table 1. Significant correlations were observed between the PSD values and psychological assessment scores; the PSD value in the delta frequency band was positively correlated with the BDI score (*r* = 0.51, *p* < 0.05) and negatively correlated with the SAI score (*r* = −0.53, *p* < 0.05). In addition, the PSD value in the theta frequency band showed a significant positive correlation with the BDI score (*r* = 0.48, *p* < 0.05).

### 3.2 Functional connectivity

**Figure 3:**
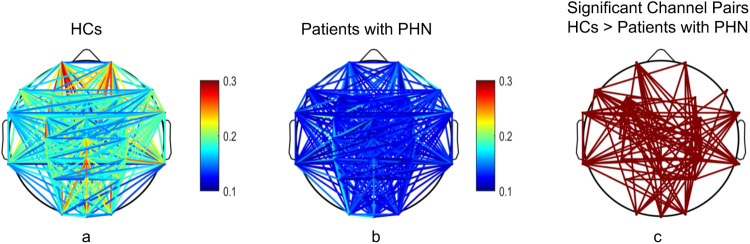
Comparison of the weighted phase lag index in the gamma frequency band between patients with PHN and HCs. **(a,b)** illustrate the functional connectivity for HCs and patients with PHN, respectively; **(c)** shows the significant channel pairs between the two groups. HC, healthy control; PHN, postherpetic neuralgia.

The wPLI values in the gamma frequency band were significantly higher in HCs compared with patients with PHN (corrected *p* < 0.05). Figure 3 illustrates the averaged wPLI for each channel pair across all participants within each group (the left and middle columns), indicating the channel pairs exhibiting significant differences (the right column), thereby emphasizing the degree of functional disconnection in patients with PHN within the gamma frequency band.

Specifically, the significant channel pairs associated with individual channels were as follows: Fp1, 9 pairs; Fp2, 5 pairs; F7, 11 pairs; F3, 14 pairs; Fz, 2 pairs; F4, 5 pairs; F8, 2 pairs; FT7, 11 pairs; FC3, 5 pairs; FCz, 5 pairs; FC4, 8 pairs; FT8, 9 pairs; C3, 5 pairs; Cz, 13 pairs; C4, 12 pairs; TP7, 12 pairs; CP3, 6 pairs; CPz, 11 pairs; CP4, 18 pairs; TP8, 10 pairs; P3, 3 pairs; Pz, 6 pairs; P4, 11 pairs; O1, 6 pairs; Oz, 6 pairs; and O2, 9 pairs.

The channel pairs exhibiting reduced connectivity were distributed across the frontal, central, parietal, and occipital regions, highlighting widespread network disruption. CP4 emerged as the most interconnected node.

## 4 Discussion

The findings of this study provide crucial insights into the neural underpinnings of PHN, demonstrating significant alterations in neural oscillations and functional connectivity in patients with the condition. These results contribute to the growing body of evidence demonstrating that chronic pain conditions such as PHN involve widespread changes in brain activity and network dynamics. The increased power across multiple EEG frequency bands and reduced functional connectivity observed in patients with PHN underscores the complexity of pain processing in the brain and suggests potential targets for neuromodulatory interventions.

### 4.1 Increased EEG power across frequency bands

One of the primary findings of this study is the elevated power observed across all the EEG frequency bands (delta, theta, alpha, beta, and gamma) in patients with PHN compared with HCs. This widespread increase in power indicates hyperactivity in the neural circuits involved in pain perception and processing. Similar results have been previously reported, reinforcing the notion that chronic pain is associated with heightened cortical excitability (10, 26).

The increase in the delta and theta frequency band power is particularly noteworthy. Delta oscillations (1–4 Hz) are typically associated with sleep, drowsiness, and low-arousal states, whereas theta oscillations (4–7 Hz) are associated with memory, emotional processing, and pain modulation (5). The elevated delta and theta frequency band power in patients with PHN suggest that these slower rhythms play a role in the sensory and affective dimensions of chronic pain. Moreover, the positive correlation between the delta and theta frequency band power and BDI scores indicates that these oscillatory changes may reflect underlying depressive symptoms, which are common comorbidities in patients with PHN.

The increased alpha and beta frequency band power further highlight the altered state of neural inhibition and sensorimotor processing in patients with PHN. Alpha rhythms (7–13 Hz) are often associated with inhibitory control and cortical idling, whereas beta rhythms (13–30 Hz) are associated with motor function and active cognitive states (27). Enhancement of these rhythms may reflect maladaptive neural mechanisms aimed at compensating for persistent pain signals, potentially contributing to sensory hypersensitivity and dysfunctional pain processing.

Gamma oscillations (30–70 Hz), which are associated with higher-order cognitive and sensory integration, were also elevated in patients with PHN. This finding aligns with previous studies that demonstrated increased gamma activity in various chronic pain conditions (10). Elevated gamma frequency band power is thought to reflect enhanced cortical excitability and increased sensory input, further supporting the notion that patients with PHN experience heightened nociceptive processing.

### 4.2 Functional connectivity deficits

In contrast to the widespread increase in EEG power, functional connectivity analysis revealed significant reductions in gamma-band connectivity among patients with PHN. The decreased wPLI values in the gamma band indicate impaired communication between the brain regions involved in pain processing and modulation. These findings are consistent with those of a previous study that demonstrated disrupted connectivity in populations with chronic pain (15).

The reduced connectivity, particularly in the CP4, Cz, and C4 channels, demonstrates their importance in sensory integration and pain processing. The CP4 region, located above the parietal cortex, is critical for sensory integration, whereas the Cz and C4 regions correspond to the central motor and somatosensory regions. The decreased connectivity between the frontal and parietal regions suggests that top-down pain signal modulation is impaired, indicating the dysfunction of sensorimotor and pain regulatory networks. Such disruptions may contribute to altered pain perception and weakened descending inhibitory control, potentially exacerbating chronic pain.

Furthermore, the significant decrease in connectivity between the frontal and parietal regions may indicate changes in the networks involved in cognitive control and pain regulation. The frontoparietal network has been linked to the top-down modulation of pain (28), and decreased connectivity in this circuit may be associated with decreased regulatory capacity. However, given that the EEG data was collected at resting state and the correlational nature of the analysis, such functional interpretations should be considered preliminary. Nonetheless, these findings indicate widespread disruptions in neural oscillatory dynamics and connectivity in PHN, suggesting potential network-level targets for future neuromodulation interventions and personalized therapies.

### 4.3 Relationship between EEG and psychological measures

A critical aspect of this study is the observed relationship between neural oscillations and the psychological measures, including those assessing depression and anxiety. The positive correlation between the delta and theta frequency band power and BDI scores suggests that slow-wave activity may serve as a neural marker of depressive symptoms in patients with PHN. This finding aligns with the broader literature suggesting shared overlapping neural mechanisms for chronic pain and depression (29).

Conversely, the negative correlation between the delta frequency band power and SAI scores indicate a potential inverse relationship between low-frequency oscillations and acute anxiety. These results reflect the distinct neural pathways underlying anxiety and depression, with anxiety being more closely associated with hyperactivity in high-frequency bands (30). By elucidating these relationships, this study highlights the need for integrated approaches to PHN management that address both the physical and psychological components of pain.

The observed relationships between the EEG PSD results and psychological assessment scores emphasize the interaction between neural activity and psychological states in PHN. Increased delta and theta frequency band power correlated with higher BDI scores, implying that low-frequency oscillations may reflect the affective dysregulation common in chronic pain. Meanwhile, the decreased gamma-band connectivity may indicate impaired sensory integration and top-down modulation, potentially adding to the physical and emotional burden of pain. These findings support a multidimensional framework for PHN, in which neural and psychological measures reflect the central and affective components of the condition. Notably, a recent review emphasized that resting-state gamma-band oscillations and connectivity disruptions are not only indicators of sensory processing but also correlate with individual pain sensitivity and emotional burden in chronic pain (31). This supports our finding that PHN-related EEG changes, particularly in the delta, theta and gamma frequency bands, may reflect a combined neurophysiological profile that includes affective symptoms and central sensitization.

### 4.4 Clinical implications and future directions

The findings of this study have several important clinical implications. First, the widespread increase in EEG power suggests that targeting hyperactive neural circuits may offer a promising avenue for neuromodulatory treatments, such as transcranial magnetic stimulation or neurofeedback. By selectively modulating the delta, theta, and gamma oscillations, these interventions can alleviate pain and improve psychological outcomes in patients with PHN. Second, the observed deficits in functional connectivity highlight the importance of restoring network-level coherence during PHN treatment. Techniques such as neurostimulation, cognitive behavioral therapy, and mindfulness-based interventions may enhance connectivity within pain-regulatory networks, promoting more effective pain modulation and reducing symptom severity.

Future research should aim to delineate the causal relationships between neural oscillations, connectivity patterns, and clinical outcomes in patients with PHN. Longitudinal studies tracking changes in EEG activity and psychological measures over time can provide valuable insights into the dynamic nature of chronic pain and its neural correlates. Additionally, expanding the sample size and incorporating multimodal imaging techniques, such as functional MRI, may enhance the generalizability and depth of these findings.

In conclusion, this study provides compelling evidence for widespread neural alterations in patients with PHN, characterized by increased EEG power and decreased functional connectivity. These findings not only advance our understanding of the neural mechanisms underlying PHN but also offer potential targets for future interventions aimed at alleviating pain and improving the quality of life in affected individuals.

## 5 Conclusion

This study provides compelling evidence of altered neural dynamics in patients with PHN, characterized by increased power across multiple EEG frequency bands and decreased functional connectivity, particularly within the gamma frequency band. These findings underscore the significant role of oscillatory neural activity and network dysfunction in the pathophysiology of chronic pain conditions, such as PHN. The observed elevations in the delta, theta, alpha, beta, and gamma frequency band power suggest widespread cortical hyperactivity and heightened sensitivity within the pain-processing circuits. The observed positive correlation between slower oscillatory activity (delta and theta frequency bands) and depressive symptoms highlights the intricate relationship between chronic pain and emotional distress, reinforcing the need for comprehensive approaches that address physical and psychological aspects of PHN management. The reduced functional connectivity in the gamma frequency band reflects impaired communication between key brain regions involved in sensory processing and pain modulation. This disrupted network integration may contribute to the persistence and intensity of pain in PHN, supporting the hypothesis that PHN is not solely a peripheral condition but also involves the central nervous system. Clinically, these results offer valuable insights for the development of targeted neuromodulatory interventions aimed at restoring normal neural oscillation patterns and connectivity.

## Data Availability

The original contributions presented in the study are included in the article/Supplementary Material, further inquiries can be directed to the corresponding author.
